# *Bordetella holmesii*: Lipid A Structures and Corresponding Genomic Sequences Comparison in Three Clinical Isolates and the Reference Strain ATCC 51541

**DOI:** 10.3390/ijms18051080

**Published:** 2017-05-18

**Authors:** Valérie Bouchez, Sami AlBitar-Nehmé, Alexey Novikov, Nicole Guiso, Martine Caroff

**Affiliations:** 1 Institut Pasteur, Unité de Prévention et Thérapies Moléculaires des Maladies Humaines, 25 rue du Dr Roux, 75724 Paris, France; valerie.bouchez@pasteur.fr (V.B.); nicole.guiso@pasteur.fr (N.G.); 2 Institute for integrative Biology of the Cell (I2BC), Commissariat à l’Energie Atomique (CEA), Centre National de la Recherche Scientifique (CNRS), Université Paris-Sud, Université Paris-Saclay, 91405 Orsay, France; sami.nehme@uniroma1.it; 3 LPS-BioSciences, I2BC, Bâtiment 409, Université de Paris-Sud, 91405 Orsay, France; alexey.novikov@lpsbiosciences.com

**Keywords:** Bordetellae, *Bordetella holmesii*, endotoxin, lipid A, structure, mass spectrometry, genomic

## Abstract

*Bordetella holmesii* can cause invasive infections but can also be isolated from the respiratory tract of patients with whooping-cough like symptoms. For the first time, we describe the lipid A structure of *B. holmesii* reference strain ATCC 51541 (alias NCTC12912 or CIP104394) and those of three French *B. holmesii* clinical isolates originating from blood (Bho1) or from respiratory samples (FR4020 and FR4101). They were investigated using chemical analyses, gas chromatography–mass spectrometry (GC–MS), and matrix-assisted laser desorption ionization–mass spectrometry (MALDI–MS). The analyses revealed a common bisphosphorylated *β*-(1→6)-linked d-glucosamine disaccharide with hydroxytetradecanoic acid in amide linkages. Similar to *B. avium*, *B. hinzii* and *B. trematum* lipids A, the hydroxytetradecanoic acid at the C-2′ position are carrying in secondary linkage a 2-hydroxytetradecanoic acid residue resulting of post-traductional biosynthesis modifications. The three clinical isolates displayed characteristic structural traits compared to the ATCC 51541 reference strain: the lipid A phosphate groups are more or less modified with glucosamine in the isolates and reference strain, but the presence of 10:0(3-OH) is only observed in the isolates. This trait was only described in *B. pertussis* and *B. parapertussis* strains, as well as in *B. petrii* isolates by the past. The genetic bases for most of the key structural elements of lipid A were analyzed and supported the structural data.

## 1. Introduction

The *Bordetella* genus contains at the moment a dozen species, of which at least five are responsible for respiratory diseases in humans and/or animals. Classical Bordetellae consist of *Bordetella pertussis*, a strict human pathogen responsible for whooping cough; *B. parapertussis* responsible for mild whooping-cough symptoms in humans, also described as a sheep pathogen; and *B. bronchiseptica*, able to infect a broad range of hosts. The Bordetellae virulence factors include toxins—such as pertussis toxin for *B. pertussis* only, adenylate cyclase-hemolysin, and lipopolysaccharide (LPS)—and adhesins—such as filamentous hemagglutinin, fimbriae and pertactin—, all involved in the binding to ciliated epithelial cells in the host upper respiratory tract. *B. bronchiseptica* and *B. pertussis* endotoxins LPS have been shown to be implicated in virulence [1,2,3,4]; therefore, it is important to compare the structures of *Bordetella* LPSs purified from other *Bordetella* pathogenic species and, particularly, from the recent *B. pertussis* relative *B. holmesii* [5].

*B. holmesii* was first described in 1995 following its isolation from the blood of a patient with septicemia [6]. At that time, this bacterium was only originating from invasive infections in immunocompromised patients. In the past years, increasing reports of the presence of *B. holmesii* in the respiratory tract of patients with pertussis-like symptoms have been published [7,8,9,10,11,12,13]. However, it is not known whether this bacterium is an opportunistic or a pathogenic one, able to induce pertussis-like symptoms in humans [14,15,16].

For the moment, it is not possible to differentiate *B. holmesii* isolates recovered from blood from isolates recovered from respiratory samples [17,18,19]. About 21 genomes of *B. holmesii* are available on The National Center for Biotechnology Information (NCBI) [20]. First considered as close to *B. pertussis* on the basis of 16S DNA analysis, *B. holmesii* is now described in the same clade as *B. hinzii*, *B. avium* and *B. trematum* on the basis of whole genome single nucleotide polymorphism (SNP)-based analysis [21]. Most virulence factors usually produced by the classical *Bordetella* seem to be missing in *B. holmesii* except a *Bordetella* master virulence regulatory system (*bvg*), a filamentous hemagglutinin (FHA)-like protein, a bvg-intermediate phaseA protein (*bipA*) ortholog, and an alcaligin operon [19,22,23,24,25]. *B. holmesii* LPS have only been roughly studied by Van den Akker in 1998 who found them phenotypically and immunologically distinct from those of *B. pertussis* [26].

We report here the detailed lipid A structures of three *B. holmesii* isolates, as compared to those of the *B. holmesii* reference strain ATCC 51541.

## 2. Results

### 2.1. Fatty Acids Composition

Total fatty acid analyses performed by gas chromatography—mass spectrometry (GC–MS) revealed the presence of 3-hydroxytetradecanoic acid [14:0(3-OH)], 2-hydroxytetradecanoic acid 14:0(2-OH), 2-hydroxydodecanoic acid 12:0(2-OH), and 3-hydroxydecanoic acid 10:0(3-OH) as well as traces of tetradecanoic acid 14:0 and dodecanoic acid 12:0 in lipids A extracted from all tested strains and isolates. They were found to be present in the relative corresponding proportions: 2.8:1:1:0.5 for ATCC51541, Bh01, and FR 4020 differing from the FR 4101 isolate having the following proportions of 2:1:1:1.2.

### 2.2. Matrix-Assisted Laser Desorption Ionization–Mass Spectrometry Structural Analyses

#### 2.2.1. Interpretation of the Main Molecular Species in the Different Lipid a Spectra

**Figure 1:**
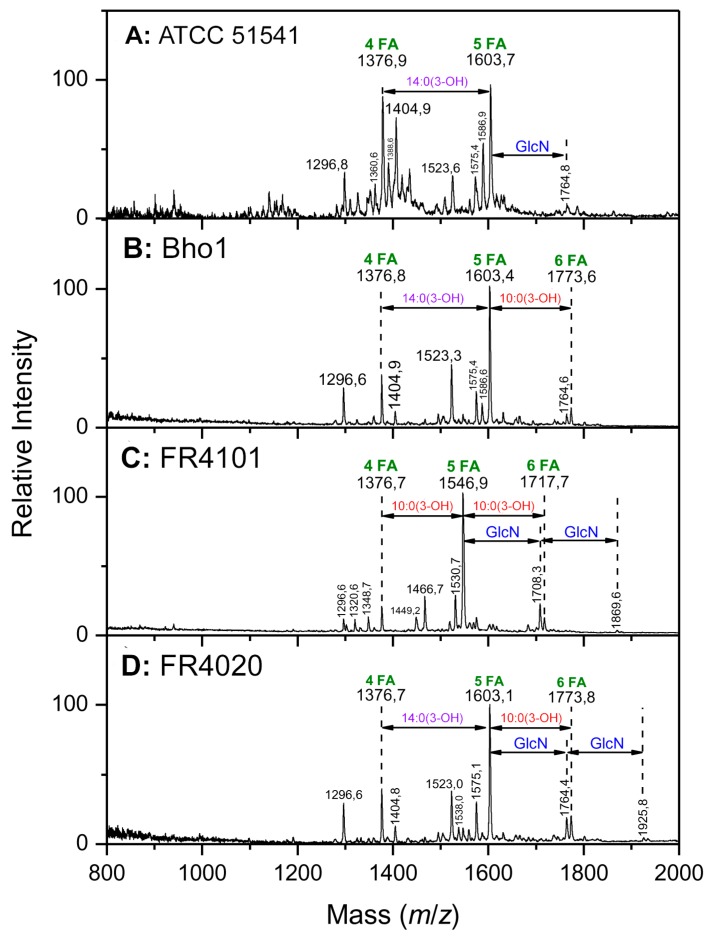
Negative-ion spectrum of lipid A from *Bordetella holmesii* strains and isolates: (**A**) ATCC 51541 reference strain; (**B**) Bho1 isolate; (**C**) FR4101 isolate; and (**D**) FR4020 isolate. FA: Fatty acid; GlcN: Glucosamine.

The negative-ion spectrum of the di-phosphoryl ATCC 51541 reference strain lipid A was heterogeneous, containing two main molecular ion signals at *m*/*z* 1376.9 and 1603.7 as illustrated in Figure 1A. Composition of the corresponding molecular species were attributed on the basis of the overall chemical composition: *m*/*z* 1603.7 would correspond to two glucosamine (GlcN), two phosphates, three 14:0(3-OH), one 14:0(2-OH), and one 12:0(2-OH); and *m*/*z* 1376.9 corresponds to *m*/*z* 1603.7 minus one 14:0(3-OH). A molecular species corresponding to *m*/*z* 1404.9 can be explained by some microheterogeneity at the level of the 12:0(2-OH) fatty acid versus the 14:0(2-OH). The same difference was observed between molecular species at *m*/*z* 1575.4 and 1603.7. Each of the molecular species presented a twin species at −16, expressing the previously described heterogeneity and peculiarity of the *Bordetella* genus, the reduced enzymes specificity at different positions carrying, in some species, 2-hydroxylated fatty acids in secondary linkage [1,27,28,29]. The latter being known as a late structural modification of the structure occurring in the membrane and leading to increased robustness of the bacterial outer membrane barrier [30].

#### 2.2.2. Distribution of the Fatty Acids on the d-Glucosamine Residues of the Clinical Isolates

The negative-ion mode matrix-assisted laser desorption ionization (MALDI) mass spectra of lipids A isolated from the three *B. holmesii* isolates displayed three major peaks at *m*/*z* 1603, 1546 and 1376 (Figure 1B–D). The first and third peaks were identical to those found in ATCC 51541 lipid A and corresponded, as explained for the reference strain, to penta- and tetra-acylated lipid A molecular species, respectively. For isolate FR4101, the ion at *m*/*z* 1546, absent in the ATCC strain spectrum, corresponded to the molecular species at *m*/*z* 1376 carrying an additional 10:0(3-OH) (170 units).

**Figure 2:**
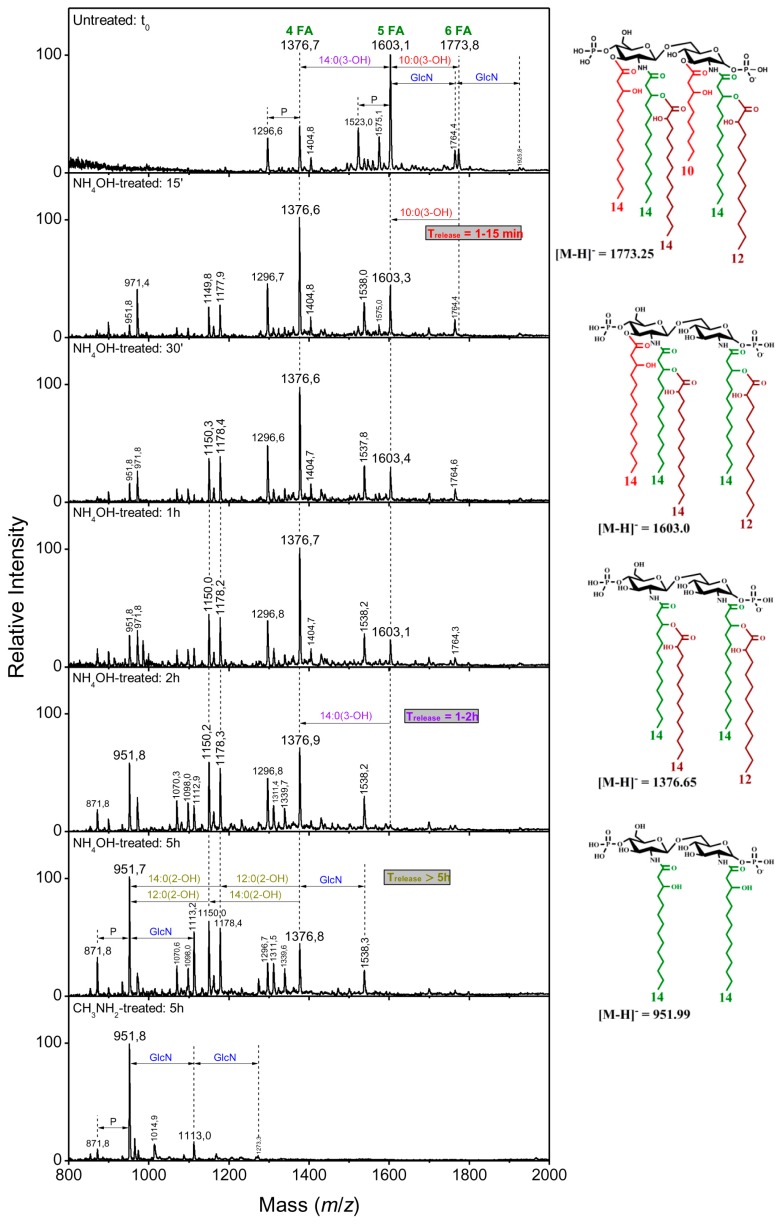
Kinetics of ester-linked fatty acids release in FR4020 in alkaline conditions and corresponding structures. Bho1 isolate and ATCC51541 behaved similarly and independently of the presence of GlcN, accordingly their kinetics are not presented here. Structures are displayed here without their GlcN substitution in order to better focus on the fatty acid pattern. However, the complete structures are presented in Figure 5.

The peaks mentioned above were surrounded by smaller ones at −16 u, such as those at *m*/*z* 1587, 1360 and 1531. These peaks in which the 16-u difference represented hydroxyl modifications, were attributed to the same structures whose secondary fatty acids linked at C-2 and C-2′, 12:0(2-OH) or 14:0(2-OH) were non-hydroxylated (14:0 or 12:0, respectively). This modification was not observed in the FR4020 lipid A mass spectrum and might be a specific trait of this isolate, with biosynthetic relevance. In addition, another type of micro-heterogeneity, regarding the length of fatty acids, was observed in the first major peaks (at *m*/*z* 1603 in Figure 1A,B,D, at *m*/*z* 1376 in Figure 1A–D and at *m*/*z* 1546 in Figure 1C) that were also doubled by minor ones at −28 u or +28 u, i.e., at *m*/*z* 1575, 1349 and 1405. The mass difference of 28 u was attributed to 2 × CH2, and related peaks were not found systematically in the three *B. holmesii* lipid A mass spectra. Of note, in the Bho1 lipid A mass spectrum, this type of heterogeneity was only observed at plus 28 u from the major peak, at *m*/*z* 1376. In the high-mass molecular ions region, two minor peaks were observed at *m*/*z* 1773 and 1764 in Bho1 (with a really minor contribution for this isolate) and FR4020 lipid A mass spectra and, at *m*/*z* 1717 and 1708 in FR4101 mass spectrum. The peak at *m*/*z* 1773, as well as the one at *m*/*z* 1717, corresponded to 10:0 (3-OH) (170 u), resulting in a hexa-acyl lipid A molecular species in Bho1 and FR4020, and FR4101, respectively. Interestingly, the mass difference between peaks at *m*/*z* 1764 and 1708 and penta-acyl lipid A molecular species at *m*/*z* 1603 and 1546.9, corresponded to 161 units for GlcN. Moreover, other two small peaks (*m*/*z* 1925 and 1869) in FR4020 and FR4101 lipid A mass spectra gave the same mass difference with *m*/*z* 1764 and 1708, suggesting that these lipid A molecular species were carrying 2 GlcN, one at each phosphate groups. This modification is described here for the fifth time in lipid A species of the *Bordetella* genus [27,31,32,33,34]. Analogous to the FR4020 mass spectrum with the exception of peaks related to GlcN addition at *m*/*z* 1538, 1869 and 1925 (Figure 2) the mass spectrum for lipid A from Bho1 displayed the same peaks as FR4020 mass spectrum. This indicates that both isolates might have similar lipid A structures with the exception that Bho1 lipid A phosphate groups displayed a lower amount of GlcN. The reference ATCC strain also showed a peak corresponding to a very small amount of GlcN, but its presence was confirmed by statistical experiments allowing to obtain persistent and slightly increased signals at this level.

#### 2.2.3. Distribution of the Fatty Acids on the d-Glucosamine Residues of the Isolates and the Reference Strain

**Figure 3:**
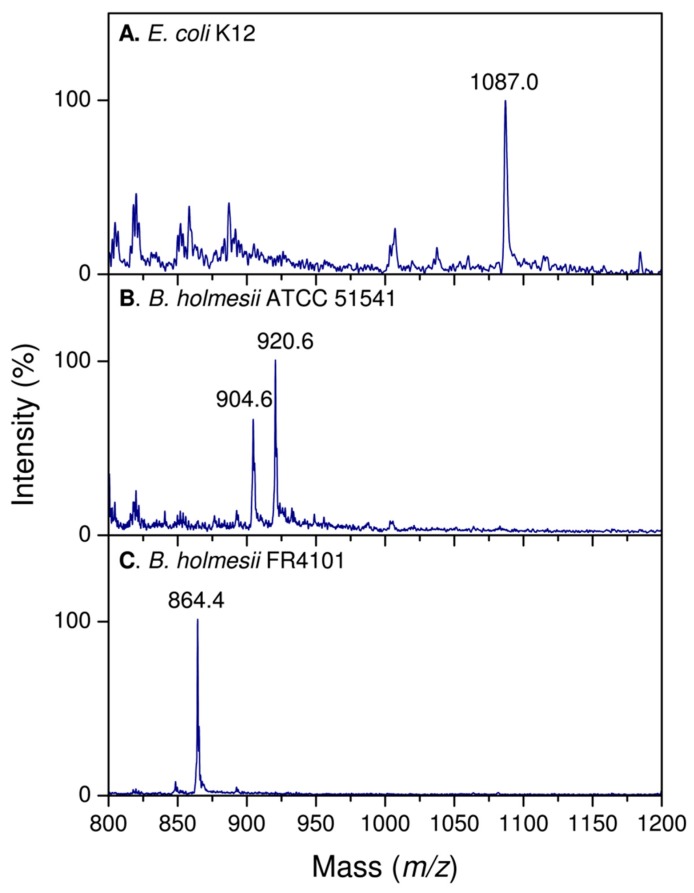
Comparison of positive-ion lipid A fragments obtained for: (**A**) *Escherichia coli* K12 taken as a reference, (**B**) *B. holmesii* ATCC 51541, and (**C**) *B. holmesii* FR4101.

The reference strain ATCC 51541 lipid A positive-ion MALDI mass spectrum presented in Figure 3 gave peaks in the lower field at *m*/*z* 920.6 and 904.6. The peak at *m*/*z* 920.6 was interpreted to correspond to an ion containing one GlcN, one phosphate, three hydroxytetradecanoic acid residues—two 14:0(3-OH) and one 14:0(2-OH)—, the second at *m*/*z* 904.6. From this fragmentation pattern, we concluded that these signals corresponded to the GlcN II (non-reducing) part of the lipids A and that the 14:0(2-OH) acid was linked at C-2′ in secondary acylation. The presence of the two peaks differing by 16 u illustrates the incomplete hydroxylation at position 2 of the 14:0 branched fatty acid.

The positive-ion MALDI mass spectra of Bho1 and FR4020 isolates also displayed a prominent fragment peak at *m*/*z* 920.6 whereas the major peak was at *m*/*z* 864.4 in the FR4101 mass spectrum. The first peak is attributed to a lipid A fragment that contained, according to the fragmentation pattern [35], the distal glucosamine (GlcN II) + one phosphate + three 14:0(OH): (two 14:0(3-OH) + one 14:0(2-OH) in secondary position). This confirms that 14:0(2-OH) was the acyloxyacyl linked at C-2′ and consequently 12:0(2-OH) is the acyloxyacyl linked at C-2 in the three isolates. Furthermore, it showed that 14:0(3-OH) is linked at C-3′ in Bho1 and FR4020. Concerning FR4101, the second peak is representing a lipid A fragment composed of GlcN II + one phosphate + one 10:0(3-OH) and two 14:0(OH) (one 14:0(3-OH) plus one 14:0(2-OH) in secondary position). This indicated that 10:0(3-OH) was ester-linked at C-3′ in GlcN II of FR4101 lipid A and confirmed that 14:0(2-OH) and 12:0(2-OH) acyl-oxy-acyls were linked at C-2′ and C-2, respectively. A small peak at *m*/*z* 694 was observed in all isolates spectra and was attributed to the lipid A fragment at 920 minus 14:0(3-OH) in Bho1, and FR4020, and minus 10:0(3-OH) in Bho1 (not shown).

#### 2.2.4. Linkage of Fatty Acids in *B. holmesii* Lipids A

**Figure 4:**
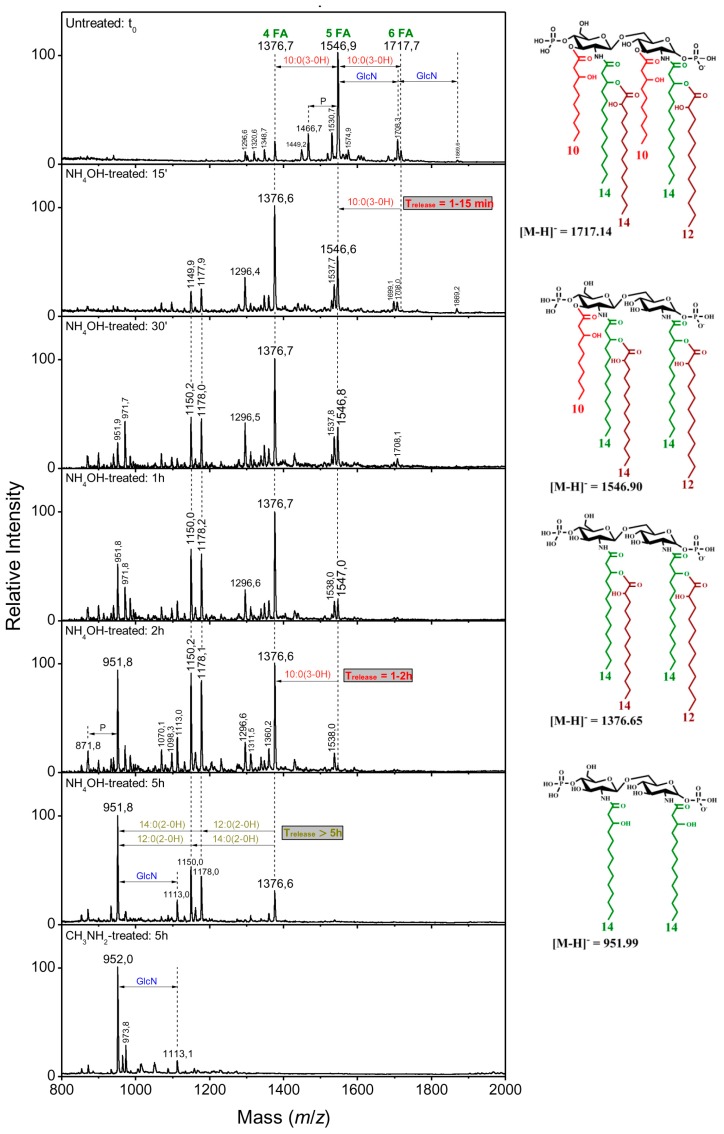
Kinetics of ester-linked fatty acid sequential release in FR4101 lipid A.

It was previously shown that sequential liberation of FAs was efficient to determine their position in the lipid A structure [32]. As shown in Table 1, the FA in ester linkages at the C-3 position are the first ones to be liberated, followed by those at C-3′, and the acyloxy-acyl secondary ester-linked FAs come as the last ones, this being due to steric hindrance conditions. Mass spectra at different NH_4_OH treatment with length times of 15 min, 30 min, 1 h, 2 h and 5 h were recorded and compared to the initial lipid A mass spectra at the starting point, t_0_ (Figure 2 and Figure 4). The partial *O*-deacylation pattern, obtained by 28% NH_4_OH treatment, was different in *B. holmesii* isolates; it was related to their linkage position. On the one hand, the time release of 10:0(3-OH) at C-3 ranged from 1 to 15 min in FR4020 and FR4101 lipids A and was illustrated by the disappearance of the hexa-acyl lipid A related peaks (at *m*/*z* 1773.8 and 1717.7 respectively) which were transformed to penta-acyl lipid A molecular species at *m*/*z* 1603.1 and 1546.9, respectively. On the other hand, the 10:0(3-OH) liberation in Bho1 took a longer time, 1–30 min (Figure 2). Then, we observed by following kinetics of sequential liberation: at the C3 position, 14:0(3-OH) was completely liberated after 1–2 h in all three clinical isolates (Figure 2 and Figure 4). This led to convert penta-acyl lipids A at *m*/*z* 1603.1 and 1546.9 to tetra-acyl ones at *m*/*z* 1376.6. In fact, two peaks at *m*/*z* 1150.2 and 1177.9 appeared after 15 min in all three clinical isolates, corresponding to *m*/*z* 1376.6 −226 u and 198 u, respectively. These mass differences were attributed to 14:0(2-OH) and 12:0(2-OH) secondary fatty acids at C-2′and C-2, respectively. Another important peak appeared at *m*/*z* 952 after 1 h in Bho1, 30 min in FR4101 and 15 min in FR4020. The structure related to this peak corresponded to a diacyl lipid A with two amide-linked acyl chains [14:0(3-OH)] at C-2 and C-2′on the lipid A core made of two GlcN and two phosphate residues.

Of particular note, the ion peak at *m*/*z* 1538 arose from addition of GlcN (161 u) to the ion at *m*/*z* 1376.6, lasting from 15 min to 5 h in FR4020 and from 15 min to 2 h in the FR4101 lipid A mass spectrum. The peak associated to the glucosamine modification was weak in the Bho1 mass spectrum, but confirmed with repeated experiments. The complete *O*-deacylation was carried out by releasing secondary ester-linked fatty acids with methylamine treatment (41% CH_3_NH_2_ at 37 °C). The negative-ion MALDI mass spectrum from Bho1 [36] displayed two peaks, after 5 h, a major one at *m*/*z* 952 (diacyl lipid A), accompanied by a minor one at *m*/*z* 872 (a mono-phosphorylated lipid A). No peak at plus GlcN was observed because of the very small amount of this molecular species present in the native molecule. In addition to these two peaks, further ones were observed in FR4101 and FR4020 mass spectra (Figure 2 and Figure 4), such as *m*/*z* 1113, which corresponded to 953 plus 161 units (GlcN) in both isolates, and a very minor not indicated peak at *m*/*z* 1274 (1113 plus 161 units), thereby demonstrating that both lipid A phosphate groups were carrying GlcN.

### 2.3. Genomic Analyses of Genes Involved in Lipid A Biosynthesis

We analyzed the sequence of genes involved in lipid A biosynthesis, more particularly focusing on the identification of the genomic basis for the two main differences observed in the lipid A structures of the three *B. holmesii* isolates in the present study, i.e., the presence/absence of glucosamines and the length of the carbon chain FA at C-3′ [14:0(3-OH) or 10:0(3-OH)]. We thus explored the Lipid A GlcN modification locus (*lgm* locus), the Acyl-[acyl-carrier-protein]--UDP-N-acetylglucosamine O-acyltransferase (*lpxA*), the Lipid A deacylase (*pagL*), the LipidA palmitoyltransferase (*pagP*), two oxygenases *lpxO1* and *lpxO2* and acyltransferase *lpxL1* genes (Table 2):

(i) An orthologous sequence corresponding to the *lgm* locus was identified in the genome of the three clinical isolates as in the ATCC 51541 reference strain (position 1966909..1970398). We observe no sequence difference for this locus comprising *ArnT* (position 1967952..1969541) between ATCC type strain and the three clinical isolates.
(ii) Bho1 and FR4020 *lpxA* sequence is identical to that of ATCC 51541 reference strain (position 3231726..3232520) but FR4101 *lpxA* sequence displays a non-synonymous SNP in position 508 leading to an amino acid modification (S instead of G in position 170 of LpxA protein). We also sequenced *lpxA* gene from 16 additional *B. holmesii* isolates collected from blood or from respiratory samples and found that this SNP was not linked to the origin of the isolates (unpublished data).
(iii) *pagL* sequence is identical for the three clinical isolates and the ATCC 51541 reference strain (position 2144078..2144623). We did not observe any sequence difference between the three tested isolates for *pagP* but a difference was observed in ATCC 51541 reference strain *pagL* sequence (position 630144..630686) corresponding to an additional G leading to a frameshift.
(iv) We found two homologs of *lpxO* in the genome of the three isolates of *B. holmesii* as in the ATCC 51541 reference strain (position 2899145..2900041 for the first one and 2402394..2403293 for the second). They respectively displayed 84% nucleotidic identity with the KF214918 *lpxO1* of *B. avium* ATCC 35086 and 84% nucleotidic identity with the KF214919 *LpxO2* of *B. avium* ATCC 35086. We then identified a *lpxL1* homolog in the three isolates as in ATCC 51541 reference strain (position 1194726..1195580) with no sequence difference between them.

## 3. Discussion

In the present study, we focused our analyses on the lipid A of different *B. holmesii* isolates originating either from blood or respiratory samples and on the ATCC 51541 reference strain.

Mass spectrometry analyses first led to the observation of a difference in the amount of modification with GlcN on the lipid A phosphate groups. The two clinical isolates FR4020 and FR4101 displayed a higher degree of modification compared to Bho1 and the ATCC strain. Such an hexosamine modification has been described in other Bordetellae species such as *B. pertussis*, *B. bronchiseptica* and *B. avium* and was attributed to a *lgm* locus encoding a glycosyl transferase *ArnT* [31,35,39]. An orthologous sequence corresponding to *lgm* locus was identified in the genome of the three clinical isolates as in the ATCC 51541 reference-strain genome with no sequence difference between them. In *B. pertussis* and *B. bronchiseptica*, the *lgm* locus has been reported to be *bvg* regulated [33]. Previous studies showed that Bho1 isolate and ATCC 51541 reference strain both have a particular non-functional *bvg* system because of a non-functional BvgA protein (due to an A insertion within *bvgA* sequence, leading to a frameshift [19,22]) that could explain why less GlcN is substituting their lipid A phosphate groups on contrary to FR4020 and FR4101. Recent sequencing and annotation of ATCC 51541 strain genome do not report this additional A within BvgA (position 3314780..3315403) suggesting that the mutation can occur frequently at random, according to subcultures or different ways of storage. Moreover, this difference is not a specific trait of respiratory samples as a functional *bvg* system is found in other *B. holmesii* isolates collected from blood [19]. All these data show that *B. holmesii* lipid A can be decorated with GlcN what has already been shown to have consequences on the modulation of host immunity via a different Toll-like receptor 4 (TLR4) activation for *B. pertussis*.

**Figure 5:**
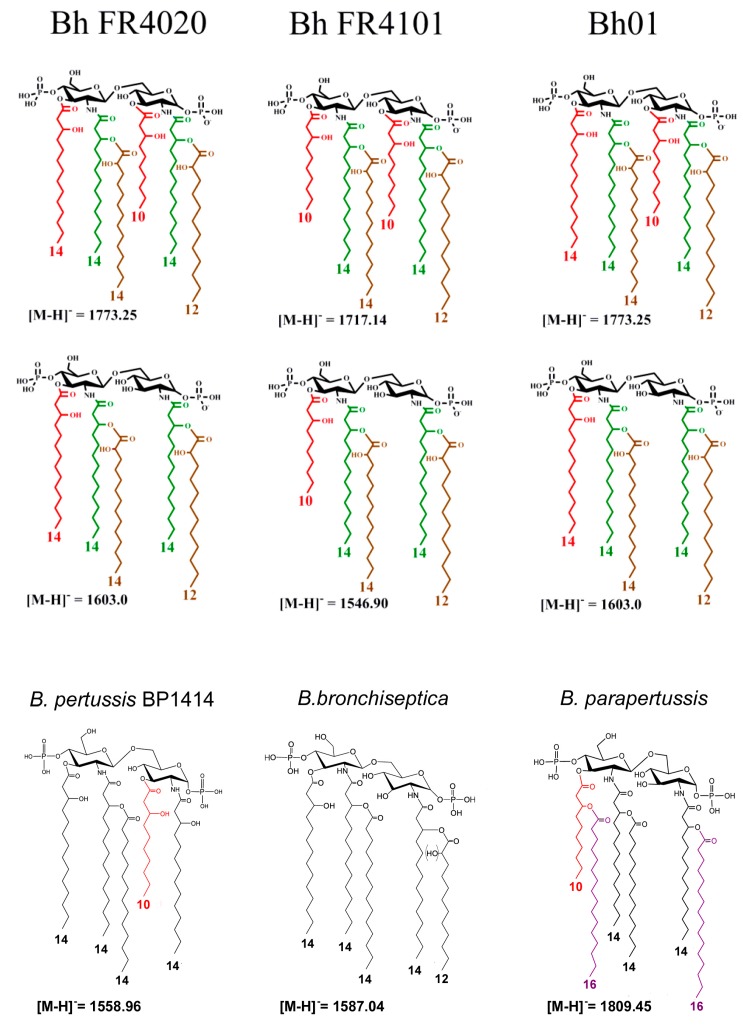
Proposed structures of the three main molecular species of higher masses present in *B. holmesii* lipid A isolates compared to those present in *B. pertussis* 1414, *B. bronchiseptica* and *B. parapertussis*.

In addition, other specificities and differences deduced from the structural analysis seem to be supported by the genomic analysis:

(i) The differences in length of the carbon FA at C3’, 14:0(3-OH) for Bho1, FR4020 and ATCC 51541 or 10:0(3-OH) for FR4101. LpxA is the first enzyme of the lipid A biosynthesis pathway catalyzing the addition of an acyl chain onto the C3’ carbon [40]. We identified *lpxA B. holmesii* gene and observed a non-synonymous SNP in FR4101, leading to an amino acid change, as compared to ATCC 51541, FR4020 and Bho1 *lpxA* sequences. Thus, we concluded that the difference observed in the length of the carbon FA at C3’ between FR4101 and the other isolates could be due to this difference within *lpxA* gene. Analysis of additional French *B. holmesii* isolates also led to the conclusion that this was not related to the blood or respiratory origin of the sample which was also confirmed by the *lpxA* sequence analysis of isolates with available genomes on NCBI.
(ii) The presence of 10:0(3-OH) at C-3, like in *B. pertussis* [1] and *B. petrii* [41] is also a common trait between the three clinical isolates. In *B. pertussis*, this is the consequence of the lack of activity of the C-3 de-*O*-acylase PagL, which has been shown to result in a lower cytokine induction capacity of the *Bordetella* human pathogens [29,42]. The presence of this fatty acid was interpreted as resulting in the facility of such bacteria, with short-chain fatty acid structures, and low acylation pattern, to escape the human host defense through the MD2–TLR4 complex [42,43]. We found that the *pagL* gene sequence is the same for the three isolates and the ATCC 51541 strain. Both H and S residues in the C-terminal part of PagL protein, described as essential for catalytic activity are present in *B. holmesii* [44]. In *B. pertussis*, *pagL* is a pseudogene because of a frameshift due to a deletion of “CA” bases. Such a deletion is not found in the *B. holmesii* sequence. Further investigations are necessary to understand why PagL is inactive in these isolates.
(iii) The presence of a secondary palmitoyl chain at two positions of lipid A (3′ and 2). We previously described the presence of such palmitoyl chains at C-3′ in *B. avium*, and in both C3′ and C2 in *B. parapertussis* [27,45] as a result of PagP action. In the present study, we observed no *pagP* sequence difference between the three tested isolates. They nevertheless display an additional G insertion as compared to ATCC 51541 reference strain, leading to a frameshift that probably explains why no palmitoyl acid is observed in these isolates.
(iv) The hydroxylation of 12:0 and 14:0 at position 2 of the two secondary acylated residues was observed in the lipid A of the three isolates (Figure 5). In Novikov et al. [27], such hydroxylations have been observed in *B. avium*, *B. hinzii* and *B. trematum* and were attributed to two homologs of the LpxO enzyme. We found two homologs of *lpxO* in the genome of the three isolates of *B. holmesii* as in the ATCC 51541 type strain that support the degree of hydroxylation observed.
(v) The presence of a 12:0(2-OH) residue in a secondary position at C-2 on the amide-linked 14:0(3-OH) is a common trait between all the *B. holmesii* structures. LpxL is a lipid A lauroyl acyltransferase. Geurtsen et al. [46] showed that LpxL1, a homolog of the classical LpxL2 lauroyl acytransferase, is present but usually poorly expressed in *B. pertussis*, compared to LpxL2 [40,46]. However, through overexpressing it in *B. pertussis* B213 strain, they showed that this enzyme led to the presence of an extra secondary 12:0(2-OH) chain at the C-2 position, like we describe here in the *B. holmesii* isolates, and they reported that this type of acylation was required for efficient infection in human macrophages and could help host infection. We found a *lpxL1* homolog in the three isolates as in ATCC 51541 type strain supporting this substitution.

All these results allowed identification of differences within lipid A structures in the different *B. holmesii* strain and isolates tested supported by both structural and genomic analysis. As illustrated in Figure 5, lipid A structures differ in their fatty acid substitution. The difference in the amount of free amino GlcN derivatives on the phosphate groups is described herein but not shown in the figure as only the peak intensity can be illustrating such differences.

## 4. Materials and Methods

### 4.1. Strains and Isolates

Strain ATCC 51541 was originating from the National Research Council (NRC) collection and was grown as previously described [47]. The three human isolates were selected from the French National Reference Center for Whooping Cough and other Bordetelloses [19]. Bho1 was isolated in 1996 from the blood of a 20 years old man with sickle-cell anemia [48]. FR4020 and FR4101 were isolated, respectively; from nasopharyngeal swabs of an adult in 2008, and of an adolescent in 2009, both presenting Pertussis-like symptoms [19,49]. Bacteria were grown on Bordet–Gengou agar (Difco by Becton Dickinson, NJ, USA) supplemented with 15% sheep defibrinated blood (Biomerieux, Marcy l’Etoile, France) at 36 °C for 72 h, plated again for 18 h and then grown in enriched Stainer Scholte medium [50].

### 4.2. LPS Preparation

LPS were extracted by the isobutyric-M ammonium hydroxide method in a 5:3 (*v/v*) ratio [51]. LPS preparations were further extracted with solvents CHCl_3_:MeOH (1:2 *v/v*) and CHCl_3_: MeOH: H_2_O (3:2:0.25, *v*/*v*/*v*) in order to remove phospholipids and lipopeptides, then treated with enzymes to remove DNA, RNA and protein contaminants, as described [32].

### 4.3. Lipid A Isolation from Whole Cells

Briefly, cells (10 mg) were washed twice with 400 µL of a fresh, single phase mixture of CHCl_3_:MeOH (1:2 *v/v*) (Sigma, St. Louis, MO, USA) and once with 400 µL of CHCl_3_:MeOH: H_2_O (3:2:0.25 *v/v/v*). The insoluble material, corresponding to washed cells, was recovered by centrifugation in the pellet, and the supernatants were discarded. The washed cells were suspended in 400 µL of isobutyric acid/ammonium hydroxide (M) (5:3 *v/v*), and kept for 1.5 h at 100 °C in a screw cap test tube under magnetic stirring. The mixture was cooled in iced water, and centrifuged (2000× *g* for 15 min). The supernatant was diluted with water (1:3 *v/v*), and lyophilized. The sample was then washed twice with 400 µL of methanol, and centrifuged (2000× *g* for 15 min). Finally, the insoluble lipid A was solubilized and extracted once with 200 µL of a mixture of chloroform: methanol:water (3:1.5:0.25 *v/v/v*) [52].

### 4.4. Lipid A Isolation from LPS as Performed by the Triethylamine Citrate (TEA) Method

A concentration of 0.01M TEA-citrate (1:1 molar ratio, pH 3.6) was used. Samples were suspended in Eppendorf^®^ tubes (Eppendorf AG, Hamburg, Germany) in the above-mentioned reagents at concentrations of 5 µg/µL, or 10 µg/µL depending to their solubility. After agitation and homogenization using an ultrasonic bath, the tubes were incubated for 1 h in a Thermomixer system (Eppendorf AG) under stirring at 1000 rpm and 100 °C [53].

### 4.5. Thin-Layer Chromatography

Thin-layer chromatography (TLC) was done on aluminum-backed silica-gel plates (Merck, Darmstadt KGaA, 64271, Germany) in the solvent [54] chloroform:methanol:water:triethylamine (12:6:1:0.04). Spots were visualized by charring after spraying with 10% sulfuric acid in ethanol for checking samples purity.

### 4.6. Fatty Acid

Fatty acids were analyzed after hydrolysis of the LPS or lipids A with 4 M HCl for 2 h at 100 °C, and neutralization, followed by treatment with 2 M NaOH 2 h at 100 °C [55], extraction with ethyl acetate, methylation of the extract with a mixture of anhydrous methanol and acetyl chloride (10:1.5 *v/v*) [56]. They were identified by GC–MS for the fine characterization and confirmation of FA composition as visualized by MALDI–MS (mass spectrometry).

### 4.7. O-Deacylation

Ester-linked fatty acids were likewise characterized after treating the lipids A with NH_4_OH during 5 h at 50 °C [32] for liberation of primary ester-linked fatty acids and with methylamine for 5 h at 50 °C for the secondary ester-linked fatty acids. GC–MS analysis of the methylated fatty acids was performed as previously described. Arachidonic acid (C_20_, Sigma), ,was used as a standard [56].

### 4.8. Mass Spectrometry

MALDI negative-ion mass spectrometry analyses were performed on a PerSeptive Voyager-DE STR model time-of-flight mass spectrometer of Applied Biosystem, (I2BC, Université de Paris Sud XI). The apparatus is equipped with a 337 nm nitrogen laser and spectra were recorded in the linear negative-ion mode with delayed extraction.

The ion-accelerating voltage was set at 20 kV. Dihydroxybenzoic acid (DHB) (Sigma) suspended in 0.1 M citric acid was used as a matrix. A few microliters of lipid A were dissolved in a mixture of chloroform:methanol:water (3:1.5:0.25 *v*/*v*/*v*) at 1 mg/mL and desalted with a few grains of ion-exchange resin (Dowex 50W-X8, Sigma, St. Louis, MO, USA) (H^+^), in an Eppendorf tube. A 1 µL aliquot of the solution (50 µL) was deposited on the target and covered with the same amount of the matrix suspended at 10 mg/mL in the same mixture of solvents [39]. Different ratios between the samples and DHB were tested when necessary. *B. pertussis* lipid A was used as an external standard.

### 4.9. Genomic Analysis

Partial genome sequences of Bho1, FR4020 and FR4101 isolates were available from Roche 454 sequencing [19]. Sequences of genes involved in lipid A biosynthesis were identified using the NCBI nucleotide and protein blast programs (blastn and blastp) (https://blast.ncbi.nlm.nih.gov). For *lgm* locus and *lpxA* gene, sequence differences were checked by polymerase chain reaction (PCR) and classical Sanger sequencing using primers Lgm-bho-F: 5′-CACATGAGCGACGAGCTCTA-3′, Lgm-bho-R: 5′-GGCTGCACTTGAACCTGTCT-3′, LpxA-bho-F: 5′-CGCACCATCTGCAAGTATCA-3′, LpxA-bho-R: 5′-CCACCATACCAATGGACAGA-3′. PCR amplifications were done from genomic DNA extracted with DNeasy Blood and Tissue kit (Qiagen, Hilden, Germany) according to the manufacturer’s instructions.

## 5. Conclusions

The *B. holmesii* lipid A structures established in the present study differ from one to the other:

(i) Presence of free amino GlcN on the phosphate groups of lipid A structure: in Bho1 as in ATCC 51541, the failure of BvgA to function leads to a weaker substitution than in the two other isolates.
(ii) Presence of 10:0(3-OH) at C-3, which we already observed in other *Bordetella* species such as *B. pertussi*s [31], is thought to be the consequence of the lack of activity of the 3 *O*-deacylase PagL, even if genomic explanation could not be found to support this hypothesis.
(iii) The 14:0(2-OH) and 12:0(2-OH) fatty acids found in all the presented structures were previously shown to be important structural features for the infection of human macrophages [42].

The combined structural virulent traits, characteristic of the *B. holmesii* lipid A structures, demonstrate the potential strength of this bacterium in its capacity to adapt for escaping to the host immune defenses. The efficiency of these traits, and especially the presence of the short-chain 10:0(3-OH) is already attested by the virulence of the whooping-cough pathogens *B. pertussis* and *B. parapertussis* [31,52] and were also recently characterized in *B. petrii* lipid A isolates [41]. However, no real difference of lipid A structure was observed between isolates from blood or respiratory origins which correlates with data from other studies [18,19]. This also correlates with the fact that, in cases of bacteremia, nasal carriage should be assessed as shown in a previous study [13].

The lipid A structures we characterized along the years on numerous strains and species always gave important information confirmed by genomics; lipid A is a powerful taxonomic tool and this work is another example of the kind. We demonstrated here the correlation between *B. holmesii* and *B. hinzii*, *B. avium* and *B. trematum* also recently shown to belong to the same clade as based on whole genome SNP-based analysis.

As already mentioned, lipid A are good bacterial markers, and their characterization in small biological samples can now help to differentiate the human *Bordetella* pathogens.

## Figures and Tables

**Table:** 

[37]	[1]	[38]

**Table 1:** Kinetics of ester fatty acids release from all isolates and ATCC strain. (**A**) Kinetics of ester fatty acids release from lipids A from *B. holmesii* strains ATCC 51541, Bho1 and FR4020 isolates. Round numbers are given for reflecting that three experiments were summarized in this table. (**B**) Kinetics of ester fatty acids release from *B. holmesii* FR4101 lipid A.

Treatment and Time Point	[M-H]^−^ Ions *m*/*z*			Fatty Acid Released	
				Type	Position
**A**					
t_0_	1376	1603	1773		
NH_4_OH, 15 min	1376	1603	1603	10:0(3-OH)	C3
NH_4_OH, 2 h	1376	1376	1376	14:0(3-OH)	C3′
NH_4_OH, 5 h	1178	1178	1178	12:0(2-OH)	secondary C2 partial release
	1150	1150	1150	14:0(2-OH)	secondary C2′ partial release
CH_3_NH_2_, 5 h	952	952	952	14:0(2-OH)	secondary C2′
				12:0(2-OH)	secondary C2
**B**					
t_0_	1376.6	1547	1717		
NH_4_OH, 15 min	1376.6	1547	1547	10:0(3-OH)	C3
NH_4_OH, 2 h	1376.6	1376.6	1376.6	10:0(3-OH)	C3′
NH_4_OH, 5 h	1178	1178	1178	12:0(2-OH)	secondary C2 partial release
	1150	1150	1150	14:0(2-OH)	secondary C2′ partial release
CH_3_NH_2_, 5 h	952	952	952	14:0(2-OH)	secondary C2′
				12:0(2-OH)	secondary C2

t_0_: Starting time point.

**Table 2:** Genomic basis for the structural differences observed within lipid A of Bho1, FR4020, FR4101 and the ATCC51541 reference strain. Genes of interest/position within ATCC51541 reference strain (Accession Number: CP007494.1)/sequence differences observed between isolates.

Genes of Interest	Position within ATCC 51541 Reference Strain (Accession Number: CP007494.1)	Sequences Differences Observed Between Isolates
***Lgm* locus**	1966909..1970398	None
***ArnT***	1967952..1969541	None
***lpxA***	3231726..3232520	No sequence differences between Bho1, FR4020 and ATCC 51541
		Non-synonymous SNP in position 508 for FR4101 (*)
***pagL***	2144078..2144623	None
***pagP***	630144..630686	No sequence differences between Bho1, FR4020 and FR4101
		Additional G compared to ATCC51541 leading to a frameshift
*lpxO1* (**)	2899145..2900041	None
*lpxO2* (***)	2402394..2403293	None
***lpxL1***	1194726..1195580	None

(*) We also sequenced *lpxA* gene from 16 additional *B. holmesii* isolates collected from blood or from respiratory samples and found that this SNP was not linked to the origin of the isolates (unpublished data); (**) 84% Nucleotidic identity with the KF214918 *lpxO1* of *B. avium* ATCC 35086; (***) 84% Nucleotidic identity with the KF214919 *LpxO2* of *B. avium* ATCC 35086.

## Abbreviations

LPS	Lipopolysaccharide
MALDI	Matrix-ssisted laser desorption
MS	Mass spectrometry
FA	Fatty acid

## References

[1] Basheer S.M., Guiso N., Tirsoaga A., Caroff M., Novikov A. (2011). Structural modifications occurring in lipid A of *Bordetella bronchiseptica* clinical isolates as demonstrated by matrix-assisted laser desorption/ionization time-of-flight mass spectrometry. Rapid Commun. Mass Spectrom..

[2] Flak T.A., Goldman W.E. (1999). Signalling and cellular specificity of airway nitric oxide production in pertussis. Cell microbiol..

[3] Preston A., Maxim E., Toland E., Pishko E.J., Harvill E.T., Caroff M., Maskell D.J. (2003). Bordetella bronchiseptica Pagp is a Bvg-regulated lipid A palmitoyl transferase that is required for persistent colonization of the mouse respiratory tract. Mol. Microbiol..

[4] Schaeffer L.M., McCormack F.X., Wu H., Weiss A.A. (2004). Interactions of pulmonary collectins with *Bordetella bronchiseptica* and *Bordetella pertussis* lipopolysaccharide elucidate the structural basis of their antimicrobial activities. Infect. Immun..

[5] Rietschel E.T., Schade U., Jensen M., Wollenweber H.W., Luderitz O., Greisman S.G. (1982). Bacterial endotoxins: Chemical structure, biological activity and role in septicaemia. Scand. J. Infect. Dis. Suppl..

[6] Weyant R.S., Hollis D.G., Weaver R.E., Amin M.F., Steigerwalt A.G., O'Connor S.P., Whitney A.M., Daneshvar M.I., Moss C.W., Brenner D.J. (1995). *Bordetella holmesii* sp. Nov., a new gram-negative species associated with septicemia. J. Clin. Microbiol..

[7] Bottero D., Griffith M.M., Lara C., Flores D., Pianciola L., Gaillard M.E., Mazzeo M., Zamboni M.I., Spoleti M.J., Anchart E. (2013). *Bordetella holmesii* in children suspected of pertussis in Argentina. Epidemiol. Infect..

[8] Rodgers L., Martin S.W., Cohn A., Budd J., Marcon M., Terranella A., Mandal S., Salamon D., Leber A., Tondella M.L. (2013). Epidemiologic and laboratory features of a large outbreak of pertussis-like illnesses associated with cocirculating *Bordetella holmesii* and *Bordetella pertussis*–Ohio, 2010–2011. Clin. Infect. Dis..

[9] Kamiya H., Otsuka N., Ando Y., Odaira F., Yoshino S., Kawano K., Takahashi H., Nishida T., Hidaka Y., Toyoizumi-Ajisaka H. (2012). Transmission of *Bordetella holmesii* during pertussis outbreak, Japan. Emerg. Infect. Dis..

[10] Miranda C., Porte L., Garcia P. (2012). *Bordetella holmesii* in nasopharyngeal samples from chilean patients with suspected *Bordetella pertussis* infection. J. Clin. Microbiol..

[11] Mooi F.R., Bruisten S., Linde I., Reubsaet F., Heuvelman K., van der Lee S., King A.J. (2012). Characterization of *Bordetella holmesii* isolates from patients with pertussis-like illness in the netherlands. FEMS Immunol. Med. Microbiol..

[12] Njamkepo E., Bonacorsi S., Debruyne M., Gibaud S.A., Guillot S., Guiso N. (2011). Significant finding of *Bordetella holmesii* DNA in nasopharyngeal samples from french patients with suspected pertussis. J. Clin. Microbiol..

[13] Nguyen L.B., Epelboin L., Gabarre J., Lecso M., Guillot S., Bricaire F., Caumes E., Guiso N. (2013). Recurrent *Bordetella holmesii* bacteremia and nasal carriage in a patient receiving rituximab. Emerg. Infect. Dis..

[14] Guiso N., Hegerle N. (2014). Other bordetellas, lessons for and from pertussis vaccines. Expert Rev. Vaccines.

[15] Pittet L.F., Posfay-Barbe K.M. (2015). *Bordetella holmesii* infection: Current knowledge and a vision for future research. Expert. Rev. Anti. Infect. Ther..

[16] Pittet L.F., Posfay-Barbe K.M. (2016). *Bordetella holmesii*: Still emerging and elusive 20 years on. Microbiol. spectr..

[17] Planet P.J., Narechania A., Hymes S.R., Gagliardo C., Huard R.C., Whittier S., Della-Latta P., Ratner A.J. (2013). *Bordetella holmesii*: Initial genomic analysis of an emerging opportunist. Pathog. Dis..

[18] Tatti K.M., Loparev V.N., Ranganathanganakammal S., Changayil S., Frace M., Weil M.R., Sammons S., Maccannell D., Mayer L.W., Tondella M.L. (2013). Draft genome sequences of *Bordetella holmesii* strains from blood (F627) and nasopharynx (H558). Genome Announc..

[19] Bouchez V., Guiso N. (2013). *Bordetella holmesii*: Comparison of two isolates from blood and a respiratory sample. Adv. Infect. Dis..

[B20-ijms-18-01080] 20.NCBI, *Bordetella pertussis* genome and annotation report.

[21] Linz B., Ivanov Y.V., Preston A., Brinkac L., Parkhill J., Kim M., Harris S.R., Goodfield L.L., Fry N.K., Gorringe A.R. (2016). Acquisition and loss of virulence-associated factors during genome evolution and speciation in three clades of *Bordetella* species. BMC Genom..

[22] Gerlach G., Janzen S., Beier D., Gross R. (2004). Functional characterization of the BvgAS two-component system of *Bordetella holmesii*. Microbiology.

[23] Diavatopoulos D.A., Cummings C.A., van der Heide H.G., van Gent M., Liew S., Relman D.A., Mooi F.R. (2006). Characterization of a highly conserved island in the otherwise divergent *Bordetella holmesii* and *Bordetella pertussis* genomes. J. Bacteriol..

[24] Hiramatsu Y., Saito M., Otsuka N., Suzuki E., Watanabe M., Shibayama K., Kamachi K. (2016). BipA is associated with preventing autoagglutination and promoting biofilm formation in *Bordetella holmesii*. PLoS ONE.

[25] Link S., Schmitt K., Beier D., Gross R. (2007). Identification and regulation of expression of a gene encoding a filamentous hemagglutinin-related protein in *Bordetella holmesii*. BMC Microbiol..

[26] Van den Akker W.M. (1998). Lipopolysaccharide expression within the genus *Bordetella*: Influence of temperature and phase variation. Microbiology.

[27] Novikov A., Shah N.R., Albitar-Nehme S., Basheer S.M., Trento I., Tirsoaga A., Moksa M., Hirst M., Perry M.B., Hamidi A.E. (2013). Complete *Bordetella avium*, *Bordetella hinzii* and *Bordetella trematum* lipid A structures and genomic sequence analyses of the loci involved in their modifications. Innate Immun..

[28] Caroff M., Aussel L., Zarrouk H., Martin A., Richards J.C., Therisod H., Perry M.B., Karibian D. (2001). Structural variability and originality of the *Bordetella* endotoxins. J. Endotoxin Res..

[29] MacArthur I., Jones J.W., Goodlett D.R., Ernst R.K., Preston A. (2011). Role of *pagl* and *lpxo* in *Bordetella bronchiseptica* lipid A biosynthesis. J. Bact..

[30] Kawasaki K. (2012). Complexity of lipopolysaccharide modifications in *Salmonella enterica*: Its effects on endotoxin activity, membrane permeability, and resistance to antimicrobial peptides. Food Res. Int..

[31] Albitar-Nehme S., Basheer S.M., Njamkepo E., Brisson J.R., Guiso N., Caroff M. (2013). Comparison of lipopolysaccharide structures of *Bordetella pertussis* clinical isolates from pre- and post-vaccine era. Carbohyd. Res..

[32] Tirsoaga A., El Hamidi A., Perry M.B., Caroff M., Novikov A. (2007). A rapid, small-scale procedure for the structural characterization of lipid A applied to *Citrobacter* and *Bordetella* strains: Discovery of a new structural element. J. Lipid Res..

[33] Marr N., Tirsoaga A., Blanot D., Fernandez R., Caroff M. (2008). Glucosamine found as a substituent of both phosphate groups in *Bordetella* lipid A backbones: Role of a BvgAS-activated Arnt ortholog. J. Bact..

[34] Geurtsen J., Dzieciatkowska M., Steeghs L., Hamstra H.J., Boleij J., Broen K., Akkerman G., El Hassan H., Li J., Richards J.C. (2009). Identification of a novel lipopolysaccharide core biosynthesis gene cluster in *Bordetella pertussis*, and influence of core structure and lipid A glucosamine substitution on endotoxic activity. Infect. Immun..

[35] Karibian D., Brunelle A., Aussel L., Caroff M. (1999). 252Cf-plasma desorption mass spectrometry of unmodified lipid A: Fragmentation patterns and localization of fatty acids. Rapid Commun. Mass Spectrom..

[36] Albitar-Nehme S. (2014). Endotoxins of the *Bordetella* Genus: Structure, Evolution and Impact on Bacterial Virulence. Ph.D. Thesis.

[37] Caroff M., Deprun C., Richards J.C., Karibian D. (1994). Structural characterization of the lipid A of *Bordetella pertussis* 1414 endotoxin. J. Bacteriol..

[38] El Hamidi A., Novikov A., Karibian D., Perry M.B., Caroff M. (2009). Structural characterization of *Bordetella parapertussis* lipid A. J. Lipid Res..

[39] Therisod H., Labas V., Caroff M. (2001). Direct microextraction and analysis of rough-type lipopolysaccharides by combined thin-layer chromatography and MALDI mass spectrometry. Anal. Chem..

[40] Shah N.R., Albitar-Nehme S., Kim E., Marr N., Novikov A., Caroff M., Fernandez R.C. (2013). Minor modifications to the phosphate groups and the C3’ acyl chain length of lipid a in two *Bordetella pertussis* strains, BP338 and 18–323, independently affect Toll-like receptor 4 protein activation. J. Biol. Chem..

[41] Basheer S.M., Bouchez V., Novikov A., Augusto L.A., Guiso N., Caroff M. (2016). Structure activity characterization of *Bordetella petrii* lipid A, from environment to human isolates. Biochimie.

[42] Marr N., Hajjar A.M., Shah N.R., Novikov A., Yam C.S., Caroff M., Fernandez R.C. (2010). Substitution of the *Bordetella pertussis* lipid A phosphate groups with glucosamine is required for robust NF-kB activation and release of proinflammatory cytokines in cells expressing human but not murine Toll-like receptor 4-MD-2-CD14. Infect. Immun..

[43] Marr N., Novikov A., Hajjar A.M., Caroff M., Fernandez R.C. (2010). Variability in the lipooligosaccharide structure and endotoxicity among *Bordetella pertussis* strains. J. Infect. Dis..

[44] Geurtsen J., Steeghs L., Hove J.T., van der Ley P., Tommassen J. (2005). Dissemination of lipid A deacylases (Pagl) among gram-negative bacteria: Identification of active-site histidine and serine residues. J. Biol. Chem..

[45] Hittle L.E., Jones J.W., Hajjar A.M., Ernst R.K., Preston A. (2015). *Bordetella parapertussis* Pagp mediates the addition of two palmitates to the lipopolysaccharide lipid A. J. Bacteriol..

[46] Geurtsen J., Angevaare E., Janssen M., Hamstra H.J., ten Hove J., de Haan A., Kuipers B., Tommassen J., van der Ley P. (2007). A novel secondary acyl chain in the lipopolysaccharide of *Bordetella pertussis* required for efficient infection of human macrophages. J. Biol. Chem..

[47] Di Fabio J.L., Caroff M., Karibian D., Richards J.C., Perry M.B. (1992). Characterization of the common antigenic lipopolysaccharide *O*-chains produced by *Bordetella bronchiseptica* and *Bordetella parapertussis*. FEMS Microbiol. Lett..

[48] Njamkepo E., Delisle F., Hagege I., Gerbaud G., Guiso N. (2000). *Bordetella holmesii* isolated from a patient with sickle cell anemia: Analysis and comparison with other *Bordetella holmesii* isolates. Clin. Microbiol. Infect..

[49] Tizolova A., Guiso N., Guillot S. (2013). Insertion sequences shared by *Bordetella* species and implications for the biological diagnosis of pertussis syndrome. Eur. J. Clin. Microbiol. Infect. Dis..

[50] Stainer D.W., Scholte M.J. (1970). A simple chemically defined medium for the production of phase I *Bordetella pertussis*. J. Gen. Microbiol..

[51] Caroff M. (2002). Brevet Français. Brevet International Patent.

[52] El Hamidi A., Tirsoaga A., Novikov A., Hussein A., Caroff M. (2005). Microextraction of bacterial lipid A: Easy and rapid method for mass spectrometric characterization. J. Lipid Res..

[53] Chafchaouni-Moussaoui I., Novikov A., Bhrada F., Perry M.B., Filali-Maltouf A., Caroff M. (2011). A new rapid and micro-scale hydrolysis, using triethylamine citrate, for lipopolysaccharide characterization by mass spectrometry. Rapid Commun. Mass Spectrom..

[54] Caroff M., Tacken A., Szabo L. (1988). Detergent-accelerated hydrolysis of bacterial endotoxins and determination of the anomeric configuration of the glycosyl phosphate present in the “isolated lipid A” fragment of the bordetella pertussis endotoxin. Carbohydr. Res..

[55] Haeffner N., Chaby R., Szabo L. (1977). Identification of 2-methyl-3-hydroxydecanoic and 2-methyl-3-hydroxytetradecanoic acids in the ‘Lipid X’ fraction of the *Bordetella pertussis* endotoxin. Eur. J. Biochem..

[56] Wollenweber H.W., Rietschel E.T. (1990). Analysis of lipopolysaccharide (lipid A) fatty acids. J. Microbiol. Methods.

